# Observing half-integer topological winding numbers in non-Hermitian synthetic lattices

**DOI:** 10.1038/s41377-025-01909-8

**Published:** 2025-06-24

**Authors:** Mu Yang, Yu-Wei Liao, Hao-Qing Zhang, Yue Li, Zhi-He Hao, Zheng-Wei Zhou, Xi-Wang Luo, Jin-Shi Xu, Chuan-Feng Li, Guang-Can Guo

**Affiliations:** 1 Laboratory of Quantum Information, University of Science and Technology of China, Hefei, 230026 China; 2 Anhui Province Key Laboratory of Quantum Network, University of Science and Technology of China, Hefei, Anhui 230026 China; 3 CAS Center For Excellence in Quantum Information and Quantum Physics, University of Science and Technology of China, Hefei, 230026 China; 4 Hefei National Laboratory, University of Science and Technology of China, Hefei, 230088 China

**Keywords:** Photonic crystals, Optical physics, Liquid crystals

## Abstract

Non-Hermitian (NH) systems have revealed unique topological phenomena that are not observed in Hermitian counterparts, such as novel topology classifications and the NH skin effect. In periodic NH systems, eigenenergies become complex and exhibit windings in the complex plane, while eigenstate winding numbers, which are strictly integers in Hermitian systems, can take half-integer values. However, direct experimental observation of NH winding of both eigenenergies and eigenstates, especially the half-integer winding, remains a significant challenge. In this work, we utilize the orbital angular momentum (OAM) synthetic dimension to construct an NH topological lattice and achieve direct observation of both eigenstate and eigenenergy windings. We report the first experimental observation of a half-integer eigenstate winding number, and reveal the intrinsic relationship between the direction in the NH skin dynamics and eigenenergy windings. Furthermore, by partitioning the OAM chain into two semi-infinite chains, we observe zero boundary modes and demonstrate that their distributions are jointly determined by the winding numbers of both the eigenstates and eigenenergies. This work provides comprehensive insights into NH topologies and offers a new experimental platform for exploring NH phenomena.

## Introduction

Recent advancements in non-Hermitian (NH) systems have unveiled new phenomena in topological matter, which lack counterparts in Hermitian systems^1–8^. For example, the number of turns that the eigenstate wavefunctions “wind” over the Brillouin zone manifold is a quantized topological invariant that governs the behavior of edge states. This principle, known as bulk-boundary correspondence, is a fundamental concept in the study of topological phases of matter. In NH system, such eigenstate winding can exhibit half-integer values^9,10^, deviating from the integer winding numbers typical of Hermitian systems. Additionally, the eigenenergies become complex and exhibit windings in the complex plane^11^ under periodic boundary conditions, which signal the presence of the NH skin effect^12^.

The half-integer eigenstate windings, together with the complex eigenenergy windings, play a crucial role in enhancing the understanding of topological properties in NH systems and in elucidating the NH bulk-boundary correspondence. However, the NH skin effect introduces extreme sensitivity to boundary conditions, leading to substantial modifications in the spectrum and the failure of conventional bulk-boundary correspondence. Under open boundary conditions, the NH bulk-boundary correspondence is appropriately described by the non-Bloch winding number^13–16^. In alternative configurations, such as semi-infinite chains that avoid two-end coupling, the characterization of edge states necessitates the use of non-Hermitian half-integer winding numbers^10,17,18^.

To date, eigenenergy windings in NH systems have been extensively studied across various platforms, including photonic systems^11,19,20^, acoustic metamaterials^21^, ion traps^22^, and mechanical membranes^7,23^. Additionally, half-integer charges in the eigenstates around exceptional points have been experimentally observed^4^. However, the eigenstate windings over the Brillouin zone manifold, which are directly associated with edge states, have only been observed in closed Hermitian systems^24,25^, where the winding numbers are strictly integer-valued. In this work, we leverage synthetic dimension^26–29^ based on the orbital angular momentum (OAM)^30,31^ to investigate the nontrivial topological windings of both eigenenergies and eigenstates in an NH topological lattice. We not only establish the intrinsic connection between eigenenergy windings and NH skin dynamics^32^, but also report, for the first time, the observation of a half-integer winding number as the eigenstates wind over the Brillouin zone. Furthermore, by partitioning the infinite OAM chain into two semi-infinite chains via a strategically designed pinhole, we engineer a system with a single boundary at the zero-OAM site, providing evidence of bulk-boundary correspondence associated with half-integer winding numbers.

## Results

### Theoretical framework

**Fig. 1:**
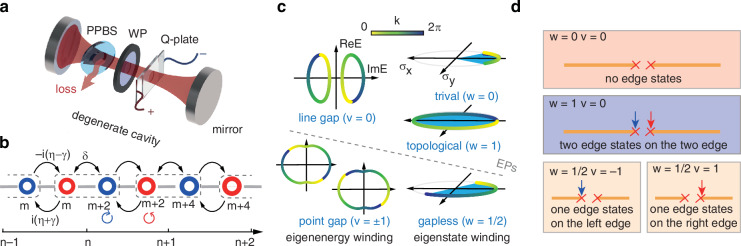
Topological features of the non-Hermitian lattice along the synthetic orbital angular momentum (OAM) dimension. **a** Experimental setup using a Q-plate, a wave plate (WP), and a partial polarising beam splitter (PPBS) in the degenerate cavity. **b** The non-Hermitian Su-Schrieffer-Heeger (SSH)-like lattice formed by different OAM modes (doughnut-shaped rings). The left ($\circlearrowleft$) and right ($\circlearrowright$) circular polarisations are labeled in red and blue, respectively. **c** Topological phases for the non-Hermitian lattice determined by the windings of the eigenstates and eigenenergies. The interface of NH topological phase transition is exceptional points (EPs). $v$ represents the eigenenergy winding number and $w$ represents the eigenstate winding number. **d** The edge states on the partitioned infinite chain are consistent with winding numbers of eigenstates and eigenenergies. The red and blue arrows represent the edge state at the left or right edge, respectively

Our experimental approach commences with the construction of a 1D NH Su-Schrieffer-Heeger (SSH) model^14,33–36^. To facilitate this, we utilize a degenerate optical cavity, as illustrated in Fig. 1a, designed to accommodate multiple OAM mode resonances (see Methods for details). Within this cavity, discrete even OAM modes ($m$) together with left ($\circlearrowleft$) and right ($\circlearrowright$) circular polarizations form a 1D lattice. Optical elements are strategically introduced into the cavity to enable tunneling along the synthetic lattice. In particular, the cavity incorporates an anisotropic liquid crystal medium known as a Q-plate to introduce intra-unit-cell hopping through the spin-orbital interaction of light^37^. The Q-plate partially transfers angular momentum between orbit and spin while preserving total angular momentum which labels the unit cell. The strength of this hopping $\delta$ is controlled by the applied electric field. Additionally, a birefringent wave plate (WP) and a partially polarized beam splitter (PPBS) within the cavity lead to the hopping between polarizations in adjacent unit cells. The coupling constant is expressed as $\pm i(\eta \pm \gamma )$, where $\eta$ represents phase difference between ordinary ray and exordinary ray passing though the WP, and $\gamma =-\mathrm{ln}P$ is the NH part controlled by the transmission reflection ratio $P$ of vertically polarized photons from the PPBS.

With this configuration, the polarised OAM modes form a 1D NH SSH-like lattice (Fig. 1b) with tight-binding approximation. The Hamiltonian can be expressed as (see Supplementary information (SI) I for details)

$$\begin{array}{c}H=-\mathop{\sum}\limits_{n}\delta ({a}_{\circlearrowright ,n}^{\dagger }{a}_{\circlearrowleft ,n}+{\rm{h}}.{\rm{c}}.)\end{array}+i\sum _{n}[(\eta +\gamma ){a}_{\circlearrowright ,n}^{\dagger }{a}_{\circlearrowleft ,n+1}-(\eta -\gamma ){a}_{\circlearrowleft ,n+1}^{\dagger }{a}_{\circlearrowright ,n}]$$

where ${a}_{\circlearrowleft (\circlearrowright ),n}^{\dagger }$ is the photon creation operators with unit-cell index $n$ and polarizations (pseudo-spins or sublattices) $\circlearrowleft$ or $\circlearrowright$. The lattice consists of $\circlearrowleft$ and $\circlearrowright$ sublattices, which fulfill the sublattice symmetry condition^38^. In the Bloch momentum ($k$) space, the NH Hamiltonian becomes $H=\sum _{k}{{\boldsymbol{a}}}_{k}^{{\boldsymbol{\dagger }}}H(k){{\bf{a}}}_{k}$, where ${{\bf{a}}}_{k}^{\dagger }=({a}_{\circlearrowleft ,k}^{\dagger },{a}_{\circlearrowright ,k}^{\dagger })$ is the Fourier transform of the creation operators, satisfying ${a}_{\circlearrowleft (\circlearrowright ),n}^{\dagger }=\sum _{k}{{\boldsymbol{a}}}_{\circlearrowleft (\circlearrowright ),k}^{{\boldsymbol{\dagger }}}{e}^{-{ikn}}$. The Hamiltonian can be written as $H(k)=-[\delta +i\left(\eta -\gamma \right){e}^{-{ik}}]{\sigma }_{+}-[\delta -i\left(\eta +\gamma \right){e}^{{ik}}]{\sigma }_{-}$, where ${\sigma }_{\pm }=({\sigma }_{x}\pm i{\sigma }_{y})/2$ and ${\sigma }_{x,y,z}$ are Pauli operators.

The eigenvalues $\pm E(k)$ appear in pairs due to sublattice symmetry, corresponding to two complex energy NH bands. The NH spectral topology is manifest in the band structures that exhibit non-trivial winding (see Fig. 1c) in $[\mathrm{Re}(E),{\rm{Im}}(E)]$ space as the momentum $k$ varies from 0 to $2\pi$. In the line-gap phase, the complex bands manifest as two separate loops. A topological invariant, defined as

$$\begin{array}{c}v={\int }_{0}^{2\pi }\frac{{dk}}{2\pi i}{\partial }_{k}{\rm{ln}}[\det H(k)-\frac{1}{2}{\rm{Tr}}(H({\rm{k}}))]\end{array}$$

captures the nature of the two-strand winding, where the eigenenergy winding number is $v=0$. Moreover, despite sharing the same morphology, the winding directions (clockwise or anticlockwise) can differ, influencing the direction of particle transport (NH skin dynamics^32^). Conversely, when the system transitions to the point-gap phase (or gapless phase)^17^, the two bands intricately winding around each other, forming a single loop with an eigenenergy winding number $v=\pm 1$. The topological phase transition from line-gap to point-gap of the complex bands occurs at EPs, where the coupling strength satisfies $|\gamma |=|\delta \pm \eta |$.

The eigenstate, as a fiber bundle over the Brillouin zone manifold, can also host non-trivial topological windings. We can define the winding number $w$ of eigenstates as

$$\begin{array}{c}w={\int }_{0}^{2\pi }\frac{{dk}}{4\pi i}{Tr}[{\sigma }_{z}{H}^{-1}(k)\frac{\partial }{{\partial }_{k}}H(k)]\end{array}$$

In the line-gap phase ($v=0$), similar with Hermitian systems, the system is divided into the topological phase ($w=1$) to the trivial phase ($w=0$), where the phase transition occurs when the intra-cell coupling character shifts from bonding ($|\eta \pm \gamma | > |\delta |$) to anti-bonding ($|\eta \pm \gamma | < |\delta |$). Upon entering the point-gap phase (${|v|}=1$), a unique NH gapless region emerges, and this new phase is marked by the fractional topological winding number^9,10^ $w=1/2$. The winding numbers of both eigenstate and eigenenergy are required to determine the NH system topologies.

As partitioning the infinite chain into two semi-infinite chains, the phase transition of topologies leads to the transition in topological edge states at the end of a semi-infinite lattices^9,11,17^. According to the phase diagrams of topological winding numbers and edge states (see SI section II for details), the nonzero eigenstate winding number ($w=1$ or $w=1/2$) determines the number of zero edge modes (two or one) while the eigenenergy winding $v$ determines their locations, as simplified in Fig. 1d. When the winding numbers are $w=1,v=0$, two zero-energy edge states manifest at the boundaries of the two semi-infinite chains, respectively. Conversely, as the winding numbers become $w=1/2,v=-1$, the intensified right-toward skin dynamics removes the edge state in the right semi-infinite chain, leaving only one edge state situated at the boundary of the left semi-infinite chain (edge state on the right chain survives if $v=1$). It’s worth noting that there would be no edge modes for the trivial phase as $v=0,w=0$.

### Experimental results

**Fig. 2:**
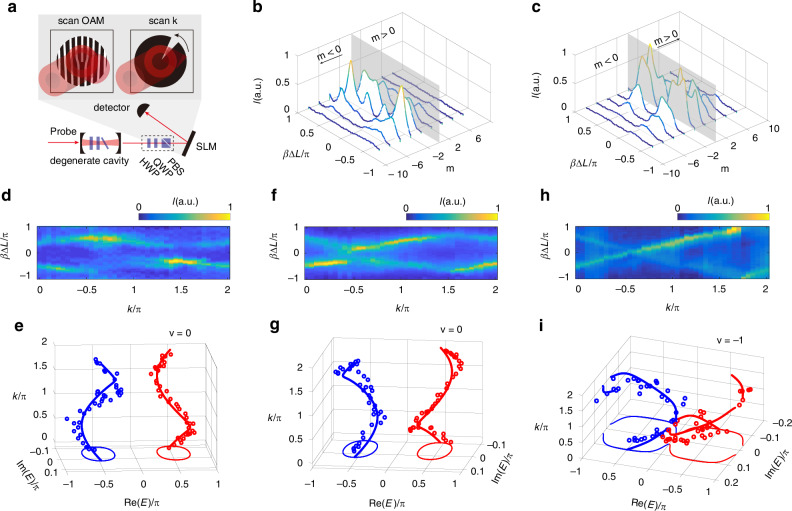
Directionalities of the photonic flow and eigenenergy windings. **a** The projection measurement on $k$ or OAM basis of transmitted photons realized by loading different phase diagrams on the spatial light modulator (SLM). The combination of quarter-wave plate (QWP), half-wave plate (HWP), and polarization beam splitter (PBS) is used for polarization projection measurement. **b**, **c** The experimental transmitted intensity spectra on OAM bases $I(m,\beta \varDelta L$) when parameters $(\gamma ,\eta ,\delta )$ are $(\mathrm{0.35,0.5}\pi ,0.2\pi )$, $(0.35,-0.5\pi ,0.2\pi )$. The gray plane corresponding to $m=0$ is on the bottom right. **d**, **f**, **h** The transmitted intensity spectra along momentum $k$ when parameters $(\gamma ,\eta ,\delta )$ are $(\mathrm{0.35,0.5}\pi ,0.2\pi )$, $(0.35,-0.5\pi ,0.2\pi )$ and $(0.35,-0.5\pi ,0.4\pi )$, respectively. **e**, **g**, **i** The complex energies in $E-k$ space extracted from **d**, **f**, and **h**. The circles represent the experimental data. The thick curves are the theoretical predictions, and the thin curves are the projections of the energies in the plane

We employ a continuous-wave (CW) probe laser to pump the cavity and subsequently detect the transmitted photon signals (see Fig. 2a). Simultaneously scanning the cavity’s length $\Delta L$, we obtain transmission intensity spectra with detuning $\beta \Delta L$, where $\beta$ represents the wave number. A spatial light modulator (SLM) is employed, configured with specific phase diagrams for projective measurement. (see section III.A and B in SI for the principle of measurement and experimental details). To explore the transport properties, a projective measurement on specific OAM bases of the output light is undertaken. The phase diagrams of a set of fork gratings are loaded on the SLM, as illustrated in Fig. 2a (Upper left). The OAM modes with corresponding OAM index can then be transformed into Gaussian modes, selectively coupled into a single-mode fiber (SMF) and subsequently detected (refer to Methods for detailed procedures). As a result, an OAM- and detuning-dependent transmitted intensity spectrum [$I(m,\beta \Delta L)$] is obtained, elucidating the optical mode distribution along the OAM lattice.

Initiating the experiment with parameters $\gamma =0.35$, $\eta =0.5\pi$ (using 3/4-WP), and $\delta =0.2\pi$, where the intra-unit-cell hopping strength ($\eta +\gamma$) from left-circular polarization to right-circular polarization is more pronounced than the reverse hopping strength ($\eta -\gamma$). This anisotropic coupling contributes to an increased population of photons evolving towards negative order OAM ($m \,<\, 0$), serving as the origins of the NH skin effect. This phenomenon is evident in the transmitted intensity spectrum with Gaussian probe laser ($m=0$) input the cavity, presented in Fig. 2b, where the photonic distribution of negative order OAM exhibits dominance characterized by slower decay and stronger intensities compared to those of positive order OAM. Conversely, a variation of $\eta$ to $-0.5\pi$ is implemented by rotating the optical axis of the WP by ${180}^{\circ }$, and the nonreciprocal intra-unit-cell hopping strength between polarizations is reversed. Consequently, the population of photons also reverses (Fig. 2c), with dominance observed in photons towards positive order OAM.

To explicate the variations in skin-induced unidirectional transport corresponding to the windings in the band structure, we then investigate the complex-band behaviors in the $E-k$ space according to the $k$-resolved transmitted intensity spectra. The reciprocal space of the OAM lattice corresponds to the wavefront azimuthal coordinate $\theta$ in the cylindrical reference frame, with the $z$ axis aligned with the beam axis. A fan diaphragm (Upper right in Fig. 2a) is constructed through phase modulation by the SLM to filter photons along the azimuthal coordinate $\theta =-k/2$, effectively isolating photons with quasi-momentum $k$ (refer to the Methods section for detailed information). In the context of the negative-toward photonic flow illustrated in Fig. 2b, the resulting transmission intensity spectrum $I(k,\beta \Delta L)$ is presented in Fig. 2d. Governed by the system’s Green function, expressed as ${G}({k},{\beta} {\Delta} {L})={\Gamma}_{0}\{{{\Gamma} -{\rm{Im}}[E(k)]+i[{\beta} {\Delta} L-{\mathrm{Re}}[E(k)]]\} }^{-1}$, the transmission intensity is denoted as $I(k,\beta \Delta L)={|G}(\beta \Delta L){|}^{2}$. Here, $\Gamma ={\Gamma }_{0}+\gamma$ with ${\Gamma }_{0}$ the loss of the empty cavity. According to the transmission intensity spectra, the real part of the complex energies, ${\mathrm{Re}}[E(k)]$, can be inferred based on the corresponding cavity detuning $\beta \Delta L$ of transmitted intensity peaks ${I}_{\max }(k)$. Conversely, the imaginary part of the complex energy, ${\rm{Im}}[E(k)]$, is approximated through the transmitted intensity, expressed as $\Gamma -{\Gamma }_{0}/\sqrt{{I}_{\max }(k)}$ (see section III.A for details). Complex energy bands are deduced and depicted in the $E$-$k$ space as dots in Fig. 2e. The observed winding of the upper (red) and lower (red) bands as two separate loops, after a $2\pi$ variation of $k$, aligns closely with theoretical predictions illustrated by thick curves. Each strand projected onto a plane forms two loops with an eigenenergy winding number of $v=0$. In contrast, in the scenario of the positive-toward photonic flow depicted in Fig. 2c, the resulting transmitted intensity spectrum is presented in Fig. 2f. The corresponding complex band in Fig. 2g maintains the two separate loop structure, retaining an eigenenergy winding number of $v=0$. However, the rotational direction of each loop changes from anticlockwise to clockwise as $k$ spans from 0 to $2\pi$, which determines the band winding direction is associated with the direction of the skin dynamics along the OAM lattice.

**Fig. 3:**
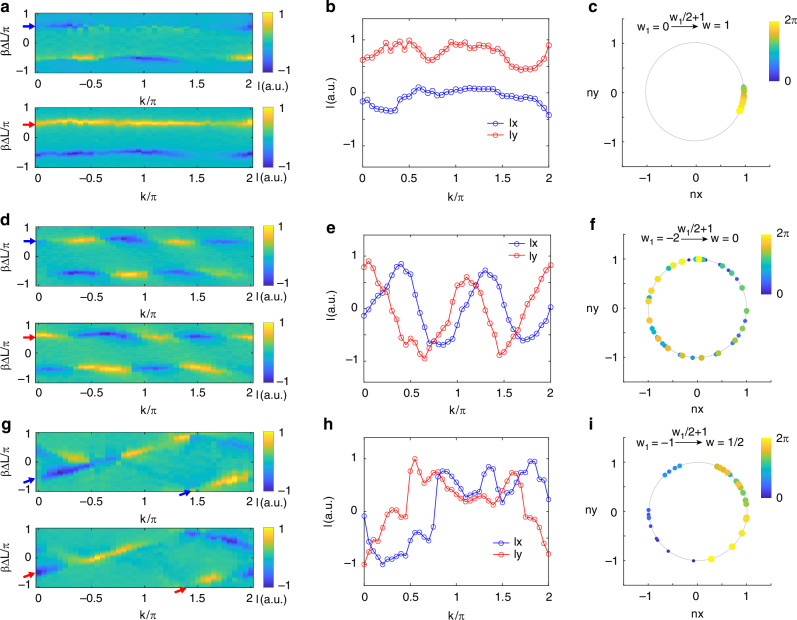
Eigenstate windings. **a**, **d**, **g** The projective transmission intensity spectra on the Pauli operators of ${\sigma }_{x}$ (top) and ${\sigma }_{y}$ (bottom) along momentum $k$ when parameters $(\gamma ,\eta ,\delta )$ are $(0.35,-0.5\pi ,0)$, $(0.35,-0.5\pi ,\pi )$ and $(0.35,-0.5\pi ,0.4\pi )$, respectively. **b**, **e**, **h** The normalized transmission intensities extracted from one of the close bands in **a**, **d**, **g**. ${I}_{x}$ (blue curves) are extracted from the band marked by the blue arrows, and ${I}_{y}$ (red curves) are extracted from the band marked by the red arrows. **c**, **f**, **i** The normalised unit vectors $[{n}_{x}(k),{n}_{y}(k)]$ as $k$ changing from 0 to $2\pi$. A reference unit circle is indicated by the gray circle

Furthermore, the determination of the system’s topological or trivial phases is facilitated through the measurement of the eigenstate winding number. The Hamiltonian of our system can be expressed in the form of spin-orbit coupling as $H(k)={n}_{x}(k){\sigma }_{x}+{n}_{y}(k){\sigma }_{y}$, where ${n}_{x}(k)$ and ${n}_{y}(k)$ represent the real and imaginary parts of non-diagonal elements. The eigenstate windings over the Brillouin zone manifold can be interpreted as the windings of the vector $[{n}_{x}(k),{n}_{y}(k)]$ in the parameter space as $k$ evolves from 0 to $2\pi$. To observe these windings, a half-wave plate (HWP), a quarter-wave plate (QWP), and a polarization beam splitter (PBS) are sequentially introduced before the SLM (Fig. 2a). This setup projects the transmitted spectra onto ${\sigma }_{x}$ and ${\sigma }_{y}$ to detect the vector $[{n}_{x}(k),{n}_{y}(k)]$. The projective transmitted intensity spectra on the Pauli operators ${\sigma }_{x}$ (top) and ${\sigma }_{y}$ (bottom) are depicted in Fig. 3a, with the parameter values $(\gamma ,\eta ,\delta )$ set at $(0.35,-0.5\pi ,0)$ and corresponding to the same phase as the parameter set $(0.35,-0.5\pi ,0.2\pi )$. Tracing one of the closed bands with $k\in (0,2\pi ]$ (indicated by the arrow in Fig. 3a), the normalized intensity ${I}_{x}(k)$ (blue, Fig. 3b) and ${I}_{y}(k)$ (red, Fig. 3b) are extracted, leading to the derivation of the associated vector $[{n}_{x}(k),{n}_{y}(k)]=[{I}_{x}(k)/\sqrt{{I}_{x}^{2}(k)+{I}_{y}^{2}(k)},{I}_{y}(k)/\sqrt{{I}_{x}^{2}(k)+{I}_{y}^{2}(k)}]$. The sublattice symmetry ensures that, the eigenstate topology can be directly obtained by only plotting the vector $[{n}_{x}(k),{n}_{y}(k)]$ in a plane (Fig. 3c) with winding number ${W}_{1}=0$ (see section III.A for details). Due to the unit cell’s dislocation and proximity to Floquet regions, the eigenstate winding number should be modified to $w={W}_{1}/2+1=1$ (see section III.C for details). Consequently, the system is conclusively situated in the topological phase. For the sake of comparison, we detected the eigenstate winding number with $(\gamma ,\eta ,\delta )$ set at $(0.35,-0.5\pi ,\pi )$, as shown in Fig. 3d–f. the eigenstate winding number is $w=0$ $({W}_{1}=-2)$, which characterizes its location in the trivial phase.

Through adjustments of the system parameters $\delta$ to $0.4\pi$ while holding other variables unchanging ($\eta =-0.5\pi$ and $\gamma =0.35\pi$), the interesting NH phase transition occurs. The corresponding transmitted intensity spectrum and the inferred complex band structure are illustrated in Fig. 2h, 2i, respectively. The measured complex band structure exhibits a non-trivial winding with an eigenenergy winding number of $v=-1$. The projected energy trajectories onto a plane form a single loop. On the other hand, the projective transmitted intensity spectra on Pauli operators ${\sigma }_{x}$ (top) and ${\sigma }_{y}$ (bottom) are depicted in Fig. 3g. Examining the winds of the unit vector $[{n}_{x}(k),{n}_{y}(k)]$ derived from the normalized intensities ${I}_{x}(k)$ and ${I}_{y}(k)$ of one closing band (Fig. 3h), we can observe the eigenstate winding number (Fig. 3i) becomes $w=1/2$ (${W}_{1}=-1$). This distinctive half-integer winding number in the NH phase, previously predicted in several theoretical studies^9,10^, is observed experimentally here for the first time. Small perturbations introduce deviations between the extracted eigenenergies (eigenvalues) and the theoretical prediction curve. However, this disorder does not impact the measurement of the winding number, as it represents a global topological invariant.

**Fig. 4:**
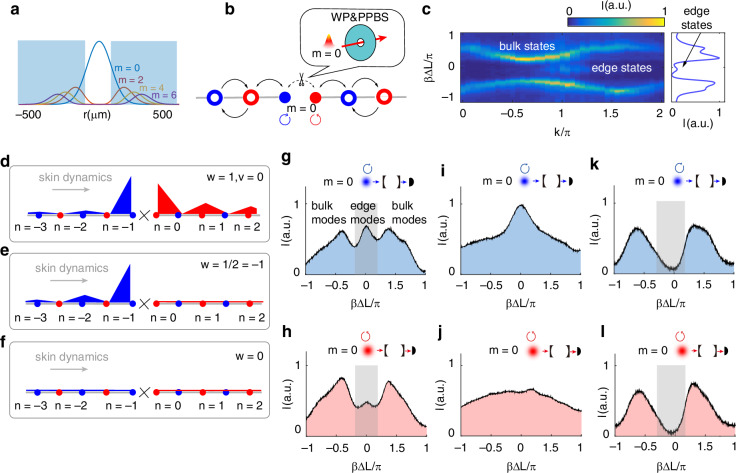
The topological edge states in the non-Hermitian OAM lattice. **a** OAM mode profile distributed relative to the pinhole, where only Gaussian mode ($m=0$) can pass through the pinhole. **b** The OAM lattice with a boundary at $m=0$ formed by the introduction of pinholes on the WP and PPBS. **c** The $k$-resolved transmitted intensity spectrum (left) and total transmitted intensity spectrum (right) as $\gamma =0$, $\delta =0.2\pi$, $\eta =0.5\pi$. **d**, **e**, **f** Theoretical edge state distributions. The parameters $(\gamma ,\eta ,\delta )$ correspond to $(0.35,-0.5\pi ,0.2\pi )$, $(0.35,-0.5\pi ,0.4\pi )$ and $(0.35,-0.5\pi ,0.9\pi )$. The eigenstate ($w$) and eigenenergy ($v$) winding numbers are labeled in the upper right corner. **g**, **i**, **k** Total transmitted intensity spectra while pumping the cavity with right-circular polarized Gaussian modes ($m=0$), respectively. The parameters $(\gamma ,\eta ,\delta )$ correspond to $(0.35,-0.5\pi ,0.2\pi )$, $(0.35,-0.5\pi ,0.4\pi )$ and $(0.35,-0.5\pi ,0.9\pi )$. The gray areas represent the zero energy gap. **h**, **j**, **l**. Total transmitted intensity spectra as the cavity is pumped with left-circular polarized Gaussian modes. The parameters $(\gamma ,\eta ,\delta )$ correspond to $(0.35,-0.5\pi ,0.2\pi )$, $(0.35,-0.5\pi ,0.4\pi )$ and $(0.35,-0.5\pi ,0.9\pi )$

To demonstrate how the topological windings relate to the boundary state, we proceed by constructing the boundary on the synthetic OAM lattice. Unlike in real space, constructing boundary states in synthetic space is not straightforward. The OAM mode ($m=0$) is the usual Gaussian mode with the maximum light intensity distributed at the center, while the high order OAM ($m\ne 0$) modes have a doughnut shape with the intensity peaks on a circle (Fig. 4a). We drill pinholes in the center of WP and PPBS via femtosecond laser ablation. Only the light with $m=0$ can pass directly through the pinhole, ensuring that the polarization remains unaffected by WP and PPBS. The polarization coupling between the $\circlearrowleft$ and $\circlearrowright$ modes at $m=0$ is effectively canceled, thereby facilitating the partitioning of the infinite OAM chain into two chains (Fig. 4b). Furthermore, the radius of OAM modes is scaled as $\sqrt{m}$. As photons propagate toward high-OAM sites, they increasingly escape the cavity without being reflected back, leading to a lack of interference with photons near $m=0$. Consequently, the edge states exhibit behavior analogous to that observed in a semi-infinite chain (see section IV in SI for details).

With this configuration, there are apparent light intensity distribution in the gap with cavity detuning $\beta \Delta L=0$, corresponding to the zeros energy boundary states (Fig. 4c, left). For briefness, we can obtain boundary states according to the intensity spectra of total light (${I}_{\text{all}}(\beta \Delta L)$). The transmitted intensity contains all components of all momentum states, thus illustrating the density of the states (DOS). As shown in Fig. 4c (right), there are two broadband strength peaks on two sides that correspond to the bulk states of the upper and lower bands, while a small peak in the center corresponds to the zero energy boundary states. Small perturbations are insufficient to close the system’s energy gap and, as such, do not affect the robustness of the edge states.

We initiate the edge state measurement with $\delta =0.2\pi$ and hold $\eta =-0.5\pi$ and $\gamma =0.35\pi$, where the winding numbers are $w=1,v=0$. Theoretical edge state distribution is illustrated in Fig. 4d. The cavity is pumped with a right-circular polarized Gaussian probe laser ($m=0,\circlearrowright$), and the zero-energy edge mode on the left chain (blue) is excited and results in a corresponding peak in the center of the gap (gray region) as depicted in Fig. 4g. Changing the probe laser to left-circular polarization excites the zero-energy edge mode on the right chain (red), leading to observable corresponding peaks (Fig. 4h). However, the peak intensities diminish due to the right-toward skin dynamics.

Upon setting $\delta$ to $0.4\pi$, transitioning the winding numbers to $w=1/2,v=-1$, the theoretical distribution of the edge state is shown in Fig. 4e. The total transmission intensity spectrum when exciting modes on the left chain (right-circular polarized laser pumping) is illustrated in Fig. 4i. The central peaks, corresponding to the edge mode, still exist. Conversely, when exciting modes on the right chain (left-circular polarized laser pumping), the central peak disappears (Fig. 4j), “dragged” away by the right-toward skin dynamics. Additionally, we extend our detection to $\delta =0.9\pi$, where the winding numbers are $w=0$. In this scenario, the topology is trivial and there is no edge state (Fig. 4f). As shown in Fig. 4k and l, no transmitted peaks in the zero-energy gap are observed.

Moreover, by reversing $\eta$ to $0.5\pi$, we achieve an eigenenergy winding of $v=1$. In this context, the reversed direction of the skin dynamics centralizes states on the right chain while simultaneously “dragging” the edge state on the left chain (see section V in SI for the extended experimental data). The observed evolution of edge states suggests that, although the existence of edge states is governed by the winding of eigenstates $w$, the specific boundary at which they manifest is determined by the winding of eigenenergies $v$.

## Discussion

In summary, we have successfully realized a non-Hermitian sublattice-symmetric SSH-like lattice incorporating an orbital angular momentum (OAM) dimension. By employing projective measurement and precise photon state readout within a degenerate cavity, we have, for the first time, directly observed a half-integer eigenstate winding number over the Brillouin zone in a non-Hermitian system. Furthermore, our experiments reveal a direct relationship between photon transport direction-rooted in the skin effect-and the winding of eigenenergies, offering promising applications for controlling the flow of light^3,39^.

By utilizing a pinhole in the optical elements, we have established a well-defined boundary, enabling the emergence of topological edge states. Our experimental results and analysis relate the edge states in semi-infinite chains to the winding of both eigenstates and eigenenergies. The edge state behaviors observed in the NH phase are consistent with the theoretical predictions associated with half-integer winding numbers.

Crucially, our investigation integrates both spectral and transport characteristics, supported by robust experimental evidence. This work lays a foundation for a unified understanding of non-Hermitian topological physics. The unprecedented control we demonstrate over spectral properties and transport/localization in this synthetic non-Hermitian system opens new avenues for exploring more exotic phenomena, such as non-Abelian physics^40^.

## Materials and methods

### The structures of the cavity

Here we consider a closed optical cavity, and the ABCD transmission matrix determines the optical OAM modes in the cavity. According to the Gaussian mode theory, the resonance condition of the cavity, which has a round trip path length $L$, is given by

$$\begin{array}{c}\beta L-(m+1)\arccos \frac{A+D}{2}=2K\pi \end{array}$$

where $\beta =2\pi /\lambda$ is the wave number and $\lambda$ is the wave length of photons in the cavity. $m$ is the OAM number and $K$ is an integer. According to Eq. 4, we can find the resonance frequency is independent with photon OAM when $A+D=2$. That’s the condition of a degenerate cavity.

The cavity contains two mirrors and two lenses with a focal length of $f=0.1$ m. The two lenses in the cavity form a 4 F telescope, allowing the transmission matrix of a round trip in the cavity satisfying degenerate condition $A+D=2$. The ratio between transmission (T) and reflection (R) for the first cavity mirror is T: R = 5:95, while T: R = 1:99 for the second mirror. These ratios are designed to satisfy the impedance-matching of the resonator. The free spectral range (FSR) of the empty cavity is about 375 MHz, and the linewidth is about 13.6 MHz.

The radius of the minimal aperture of the optical elements in the cavity is ${r}_{m}=0.25{mm}$ and the radius of pumping Gaussian modes ${r}_{0}$ is about 80*μ*m, so the maximal topological charge satisfies $m=2{r}_{m}^{2}/{r}_{0}^{2}\approx 1.95\,\times {10}^{3}$. The length of the synthetic dimension is about $1.95\,\times {10}^{3}$, which approximates an infinite chain.

### *k* projective measurements

The light field of $m$-th OAM modes can be approximately expressed as $A{e}^{{im}\theta }$, where $\theta ={\tan }^{-1}(y/x)$ with cartesian coordinates $(x,y)$ and $A$ is the amplitude. Thus, the wave functions for the $n$-th unit cell are

$$|{{n}},\circlearrowleft {{\rangle }}=A{e}^{i2n\theta }\left|\circlearrowleft \right\rangle {{;}}|{{n}},\circlearrowleft {{\rangle }}=A{e}^{i2\left(n+1\right)\theta }\left|\circlearrowright \right\rangle$$

Then, the Bloch modes can be written as

$$\begin{array}{ccc}|{k}_{0},\circlearrowleft {{\rangle }} & =\mathop{\sum}\limits_{n}{e}^{{{ink}}_{0}}{|n},\circlearrowleft {{\rangle }}={e}^{{in}{k}_{0}+i2n\theta }\left|\circlearrowleft \right\rangle & \\ & =A\delta ({k}_{0}+2\theta )\left|\circlearrowleft \right\rangle & \end{array}$$

and

$$\begin{array}{ccc}|{k}_{0},\circlearrowright {{\rangle }} & =\mathop{\sum}\limits _{n}{e}^{{{ink}}_{0}}{|n},\circlearrowright {{\rangle }}=\mathop{\sum}\limits _{n}A{e}^{{in}{k}_{0}+i(2n+2)\theta }\left|\circlearrowright \right\rangle & \\ & =A{e}^{-i{k}_{0}}\delta ({k}_{0}+2\theta )\left|\circlearrowright \right\rangle & \end{array}$$

which means the photons with Bloch momentum ${k}_{0}$ component correspond to the photons along wavefront azimuth $-\theta ={k}_{0}/2$. To isolate the ${k}_{0}$ component, here we design a phase modulation (transmittance function is $T={e}^{2\pi {iH}(x,y)}$) with a fan phase hologram

$$H(x,y)=\left\{\begin{array}{ll}\mathrm{mod}(\frac{2\pi x}{\Lambda },2\pi ), & -\theta \in \left[\frac{{k}_{0}-\Delta k}{2},\frac{{k}_{0}+\Delta k}{2}\right]\\ 0, & -\theta \,\notin \,\left[\frac{{k}_{0}-\Delta k}{2},\frac{{k}_{0}+\Delta k}{2}\right]\end{array}\right.$$

where $\Lambda$ is the grating period and $\Delta k$ the resolution of the momentum. After the transmitted light is modulated, the light with ${k}_{0}$ momentum component (near wavefront azimuth $-\theta ={k}_{0}/2$) will be modulated by a blazed grating and diffracted to +1 order position, spatially separated with the light in other parts located in 0 order diffraction position. The +1 order diffraction light can be filtered out through a slit and detected by a photodetector. Worthy to note decreasing the $\Lambda$ can increase the separation between 0 and +1 order diffraction light but will reduce the diffraction efficiency. Scanning the parameter ${k}_{0}$ from 0 to $2\pi$ and sorting the transmitted intensity spectra column by column, we can get the $k$-resolved light field intensity ${I}_{k}$.

### OAM projective measurements

To isolate (${m}_{0}$ th) OAM components in the transmitted light, we change the phase hologram to a phase fork grating, denoted as

$$\begin{array}{c}H(x,y)=\mathrm{mod}(\frac{2\pi x}{\Lambda }-{m}_{0}\theta ,2\pi )\end{array}$$

This phase hologram contains a blazed grating with period $\Lambda$, which enables the modulated light concentrated at +1 order diffraction position and spatially separated from the unmodulated light at 0 order diffraction position. Then one can filter out and collect the +1 order diffraction light through a single-mode fiber (SMF). A vortex phase ${e}^{-i{m}_{0}\theta }$ is introduced into the modulated light that the $m$-th OAM modes in the transmitted light will shift to $m-{m}_{0}$. Only the ${m}_{0}$-th OAM modes can be converted to Gaussian modes ($m=0$), which can be coupled into the SMF and detected by the photodetector. As we change the integer parameter ${m}_{0}$ of the phase hologram, the transmitted intensity spectra of different OAM components ${I}_{{OAM}}$ can be detected respectively.

## Supplementary information

Supplementary Information for ``Observing Half-Integer Topological Winding Numbers in Non-Hermitian Synthetic Lattices''

## Data Availability

All data needed to evaluate the conclusions in the paper are present in the paper and/or the Supplementary Materials.
