# Supplementary material for: Observing half-integer topological winding numbers in non-Hermitian synthetic lattices
Source: Light Sci Appl. 2025 Jun 24;14:225. doi: 10.1038/s41377-025-01909-8 (PMC12187937; doi:10.1038/s41377-025-01909-8)
Supplement: Supplementary file 1 — Supplementary Information for ``Observing Half-Integer Topological Winding Numbers in Non-Hermitian Synthetic Lattices'' [file 41377_2025_1909_MOESM1_ESM.pdf]

Supplementary Information for "Observing Half-Integer Topological Winding Numbers in Non-Hermitian Synthetic Lattices"

Mu Yang,[*] Yu-Wei Liao[*], Hao-Qing Zhang[*], Yue Li, Zhi-He Hao, Zheng-Wei Zhou, Xi-Wang Luo,[†] Jin-Shi Xu,[‡] Chuan-Feng Li,[§] and Guang-Can Guo

CAS Key Laboratory of Quantum Information,

University of Science and Technology of China, Hefei 230026, China

Anhui Province Key Laboratory of Quantum Network,

University of Science and Technology of China, Hefei, Anhui 230026, China

CAS Center For Excellence in Quantum Information and Quantum Physics,

University of Science and Technology of China, Hefei 230026, China and

Hefei National Laboratory, University of Science and Technology of China, Hefei 230088, China

CONTENTS

I. Forming non-Hermitian lattice with synthetic OAM dimension 2

II. Phase diagrams of the non-Hermitian OAM lattice 5
A. Phase diagram of eigenenergy winding numbers 5
B. Phase diagram of eigenstate winding numbers 7
C. Phase diagram of the tight binding semi-infinity lattice 8

III. Experimental details 10
A. Input and output relations of the cavity 10
B. Details of the experimental settings 12
C. Modification of the experimental eigenstate winding number 14

IV. Semi-infinite chain approximation 15

V. Extended experimental results 18

References 19

I. FORMING NON-HERMITIAN LATTICE WITH SYNTHETIC OAM DIMENSION

The first proposal of photonic synthetic dimensions starts in 2015 based on synthetic orbital angular momentum[10], and then abundant theoretical and experimental works of synthetic OAM lattice are implemented to study topological physics[20,24,27-30]. In this section, we describe how to in introduce the skin effect in a synthetic OAM dimension and form a non-Hermitian SSH-like Hamiltonian.

We consider there is a Q-plate in a closed *standing wave degenerate cavity* to couple the neighboring OAM modes, the operation of the Q-plate on the photons is

$$J_Q = \sum_n \cos(\delta/2)(a^\dagger_{\circlearrowleft,n} a_{\circlearrowleft,n} + a^\dagger_{\circlearrowright,n} a_{\circlearrowright,n}) + i\sin(\delta/2)(a^\dagger_{\circlearrowright,n-1+q} a_{\circlearrowleft,n} + \text{h.c.}), \tag{S1}$$

where the $a^\dagger_{\circlearrowleft(\circlearrowright),n}$ and $a_{\circlearrowleft(\circlearrowright),n}$ present the creation and annihilation operators of the photons in left (right) -circular polarization with total angular momentum $\hbar(2n+1)$. Notice that the spin angular momentum of left-circular $\circlearrowleft$ and right-circular $\circlearrowright$ photon are $\pm\hbar$, respectively, so the corresponding OAM number m for $a_{\circlearrowleft,n}$ and $a_{\circlearrowright,n}$ are $m = 2n$ and $m = 2n+2$, respectively. Q-plate couples the spin with orbit angular momentum, which partially changes the spin angular momentum by $\mp 2\hbar$ and the orbit angular momentum by $\pm 2q\hbar$ simultaneously. The coupling strength is $\sin\delta$, where δ is a parameter controlled by the applied

electric field. In our experiment, the parameter q is $q = 1$, that the total angular momentum is preserved by the Q-plate and only even-order OAM modes are involved.

Furthermore, to couple the polarisation of the same OAM modes, we need to introduce a wave plate (WP) in the degenerate cavity. WP only partially change the spin angular momentum and maintain OAM state, so the total angular momentum is also changed. The operation of the WP is

$$J_W = \sum_n \cos(\eta/2)(a^\dagger_{\circlearrowright,n} a_{\circlearrowleft,n} + a^\dagger_{\circlearrowleft,n} a_{\circlearrowright,n}) + i\sin(\eta/2)(e^{-i\xi} a^\dagger_{\circlearrowright,n} a_{\circlearrowleft,n+1} + e^{i\xi} a^\dagger_{\circlearrowleft,n+1} a_{\circlearrowright,n}) \tag{S2}$$

where the coupling strength η represents the phase delay between the ordinary light and extraordinary light, which is controlled by the thickness and birefringence dispersion of the WP. And the parameter ξ is the angle of the optical axis. Rotation of the optical axis can be regarded as changing the coupling $\sin\eta$ to $-\sin\eta$, which can be used to control the direction of mode hopping.

The operations of Q-plate and WP are Hermitian. To introduce the non-Hermitian item, we introduce a partially polarised beam splitter (PPBS) into the degenerate cavity. The PPBS has a high permeability ($P_H \approx 1$) for horizontally polarized light $((|\circlearrowright\rangle + |\circlearrowleft\rangle)/\sqrt{2})$ and a high permeability (P_V<1) for the vertically polarized light $((|\circlearrowright\rangle - |\circlearrowleft\rangle)/\sqrt{2})$. Similar to the WP, the PPBS also preserves the OAM, so the action of PPBS is

$$J_M = e^{-\frac{\gamma}{2}} \sum_n \cos(\frac{i\gamma}{2})(a^\dagger_{\circlearrowleft,n} a_{\circlearrowleft,n} + a^\dagger_{\circlearrowright,n} a_{\circlearrowright,n}) - i\sin(\frac{i\gamma}{2})(a^\dagger_{\circlearrowright,n} a_{\circlearrowleft,n+1} + \text{h.c.}), \tag{S3}$$

where $\gamma = -\ln\sqrt{P_V/P_H}$ is the non-Hrmitian coupling strength. Worthy to note $e^{-\frac{\gamma}{2}}$ is a global loss of the cavity, which will only influence the line width of the transmitted spectra. And here we redefine the operation $J_M/e^{-\frac{\gamma}{2}} \to J_M$.

To theoretically analyse the properties of the system, we simplify the system with tight-binding approximation, where the coupling strength δ, η, γ are small. For $\cos\delta \approx 1$ and $\sin\delta \approx \delta$, the operation of Q-plate becomes

$$J_Q \approx 1 + i\sum_n \frac{\delta}{2}(a^\dagger_{\circlearrowright,n} a_{\circlearrowleft,n} + \text{h.c.}) = e^{i\sum_n \frac{\delta}{2}(a^\dagger_{\circlearrowright,n} a_{\circlearrowleft,n} + \text{h.c.})}. \tag{S4}$$

Similarly, the operations of WP ($\xi = \pi/2$) and PPBS can be written as

$$J_W \approx 1 + \sum_n \frac{\eta}{2}(a^\dagger_{\circlearrowright,n} a_{\circlearrowleft,n+1} - a^\dagger_{\circlearrowleft,n+1} a_{\circlearrowright,n}) = e^{\sum_n \frac{\eta}{2}(a^\dagger_{\circlearrowright,n} a_{\circlearrowleft,n+1} - a^\dagger_{\circlearrowleft,n+1} a_{\circlearrowright,n})}, \tag{S5}$$

and

$$J_M \approx 1 + \sum_n \frac{\gamma}{2}(a^\dagger_{\circlearrowright,n} a_{\circlearrowleft,n+1} + \text{h.c.}) = e^{\sum_n \frac{\gamma}{2}(a^\dagger_{\circlearrowright,n} a_{\circlearrowleft,n+1} + \text{h.c.})}. \tag{S6}$$

In a round trip, the light passes Q-plate, WP, and PPBS respectively, and then returns. The evolution of the light field can be expressed as

$$\begin{aligned}\hat{U} &= J_Q J_W J_M J_M J_W J_Q \\ &= e^{i\sum_n \delta(a^{\dagger}_{\circlearrowright,n} a_{\circlearrowleft,n} + \text{h.c.}) + \sum_n [(\eta+\gamma) a^{\dagger}_{\circlearrowright,n} a_{\circlearrowleft,n+1} - (\eta-\gamma) a^{\dagger}_{\circlearrowleft,n+1} a_{\circlearrowright,n}]}.\end{aligned} \tag{S7}$$

We can define the Hamiltonian, satisfying $\hat{U} = e^{-i\hat{H}}$. And we can get a one-dimensional (1D) non-Hermitian Su-Schrieffer-Heeger (SSH)-like Hamiltonian, which is given by

$$\hat{H} = -\sum_n \delta(a^{\dagger}_{\circlearrowright,n} a_{\circlearrowleft,n} + \text{h.c.}) + i\sum_n [(\eta+\gamma) a^{\dagger}_{\circlearrowright,n} a_{\circlearrowleft,n+1} - (\eta-\gamma) a^{\dagger}_{\circlearrowleft,n+1} a_{\circlearrowright,n}]. \tag{S8}$$

The coupled OAM lattice has spatial translational symmetry, so we can introduce a Bloch modes $|k\rangle = \sum_n e^{ink}|n\rangle$ along OAM lattices in quasi-momentum space. The operation of the Q-plate can be recast in momentum k-space as as the spin-orbit coupling (SOC) form $J_Q = \sum_k \mathbf{a}^{\dagger}_k J_Q(k) \mathbf{a}_k$, where $\mathbf{a}^{\dagger}_k = (a^{\dagger}_{\circlearrowleft,k}$, $a^{\dagger}_{\circlearrowright,k})$ is the Fourier transform of the annihilation operators, satisfying

$$\begin{aligned} a_{\circlearrowright,n} = \frac{1}{\sqrt{N}} \sum_k a_{\circlearrowright,k} e^{ikn};\ a_{\circlearrowleft,n} = \frac{1}{\sqrt{N}} \sum_k a_{\circlearrowleft,k} e^{ikn}, \\ a^{\dagger}_{\circlearrowright,n} = \frac{1}{\sqrt{N}} \sum_k a^{\dagger}_{\circlearrowright,k} e^{-ikn};\ a^{\dagger}_{\circlearrowleft,n} = \frac{1}{\sqrt{N}} \sum_k a^{\dagger}_{\circlearrowleft,k} e^{-ikn}, \end{aligned} \tag{S9}$$

where N is the number of lattice sites. Going to the k-space, the Hamiltonian in momentum space is

$$\hat{H}(k) = \sum_k \begin{pmatrix} a^{\dagger}_{\circlearrowleft,k} & a^{\dagger}_{\circlearrowright,k} \end{pmatrix} \begin{pmatrix} 0 & -\delta - i(\eta-\gamma)e^{-ik} \\ -\delta + i(\eta+\gamma)e^{ik} & 0 \end{pmatrix} \begin{pmatrix} a_{\circlearrowleft,k} \\ a_{\circlearrowright,k} \end{pmatrix}, \tag{S10}$$

and we can easily get the eigenenergy, denoted as

$$E^{\pm}(k) = \pm\sqrt{\eta^2 + \delta^2 - \gamma^2 + 2\delta\eta\sin k - 2i\delta\gamma\cos k} \tag{S11}$$

In the parameter space, there are points that two eigenvalues collapse ($\text{Re}(E) = \text{Im}(E) = 0$), named as exceptional points (EPs), which satisfies

$$\eta = \begin{cases} -\delta \pm \gamma, & k = \pi/2, \\ \delta \pm \gamma, & k = -\pi/2. \end{cases} \tag{S12}$$

II. PHASE DIAGRAMS OF THE NON-HERMITIAN OAM LATTICE

A. Phase diagram of eigenenergy winding numbers

According to the complex energy behaviors, there exists a method to characterize different phases of the system. One can define the integer topological invariant as

$$\begin{aligned} v &= \int_0^{2\pi} \frac{dk}{2\pi i} \partial_k \ln\{\det \hat{H}(k) - \frac{1}{2}\mathrm{Tr}[\hat{H}(k)]\} \\ &= \frac{1}{2\pi i}\int_{BZ} \frac{dE^+}{E^+} + \frac{1}{2\pi i}\int_{BZ} \frac{dE^-}{E^-}. \end{aligned} \tag{S13}$$

Geometrically, such braiding invariance v describes how many times two bands braid with each other and the sign for the direction of the braiding. $E^{\pm}(k)$ have crossing points with real and imaginary axes when $[E^{\pm}(k)]^2 \in \mathbb{R}$, especially cross real axes ($\mathrm{Im}[E^{\pm}(k)] = 0$) when $[E^{\pm}(k)]^2 > 0$ and imaginary axes ($\mathrm{Re}[E^{\pm}(k)] = 0$) when $[E^{\pm}(k)]^2 < 0$. We now discuss the braiding under different parameters:

- When $\gamma > |\delta - \eta|$ and $\gamma > |\delta + \eta|$, $E^{\pm}(k)$ both have two crossing points with imaginary axes at $k = \pi/2$ and $k = 3\pi/2$ while don't have crossing point with imaginary axes. This means $E^{\pm}(k)$ don't enclose the point $E = 0$ for $k \in [0, 2\pi]$ and $v = 0$ based on residue theorem. The complex energies of Eq. (S11) form two independent loops in [Re(E), Im(E)] space as k changing from 0 to 2π.

- When $\gamma < |\delta - \eta|$ and $\gamma < |\delta + \eta|$, $E^{\pm}(k)$ both have two crossing points with imaginary axes at $k = \pi/2$ and $k = 3\pi/2$ while don't have crossing point with real axes. Thus $E^{\pm}(k)$ don't enclose the point $E = 0$ for $k \in [0, 2\pi]$ and $v = 0$. The complex energies of Eq. (S11) form two independent loops in [Re(E), Im(E)] space as k changing from 0 to 2π.

- When $\gamma > |\delta - \eta|$ and $\gamma < |\delta + \eta|$, $E^{\pm}(k)$ have one crossing point with real axes at $k = \pi/2$ and one crossing point with imaginary axes at $k = 3\pi/2$. The complex energies form a loop as k changing from 0 to 2π. We can define a 4π-periodic piecewise function $f(\tilde{k})$ describing the continuous loop:

$$f(\tilde{k}) = \begin{cases} \sqrt{(\delta\cos\tilde{k} - i\gamma)^2 + (\delta\sin\tilde{k} + \eta)^2} & \tilde{k} \in \left[0, \frac{3\pi}{2}\right) \\ -\sqrt{(\delta\cos\tilde{k} - i\gamma)^2 + (\delta\sin\tilde{k} + \eta)^2} & \tilde{k} \in \left[\frac{3\pi}{2}, \frac{7\pi}{2}\right) \\ \sqrt{(\delta\cos\tilde{k} - i\gamma)^2 + (\delta\sin\tilde{k} + \eta)^2} & \tilde{k} \in \left[\frac{7\pi}{2}, 4\pi\right) \end{cases} \tag{S14}$$

and then the braiding degree becomes:

$$v = \frac{1}{2\pi i}\int_0^{4\pi} d\tilde{k} \frac{d}{d\tilde{k}} \ln f(\tilde{k}). \tag{S15}$$

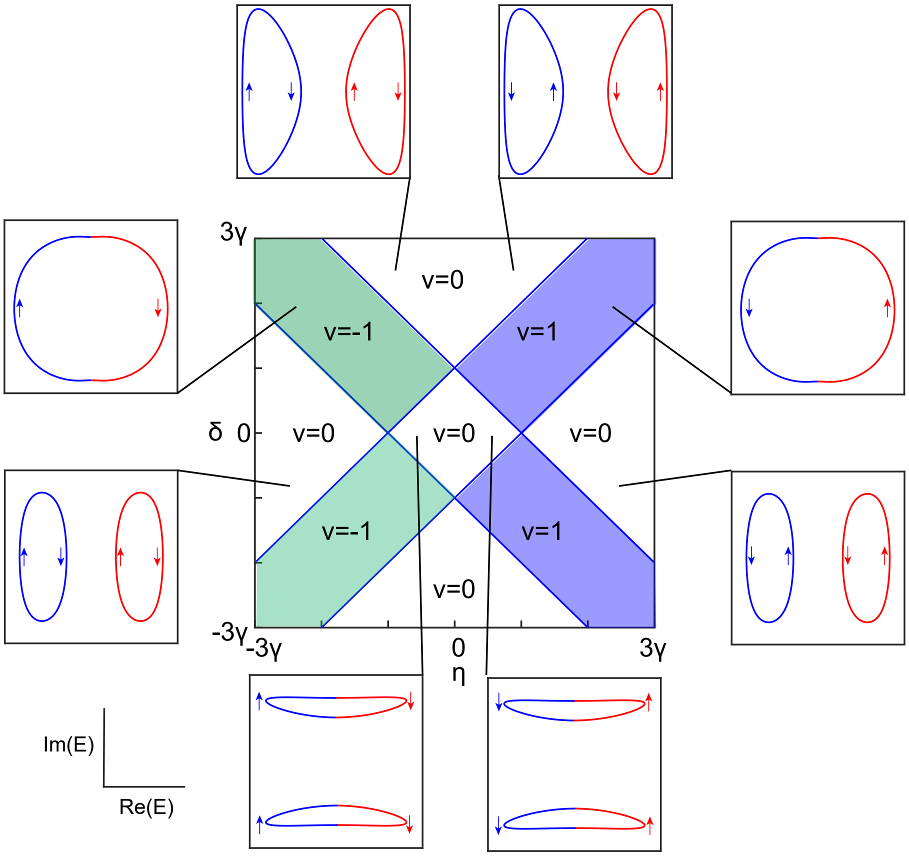

Figure S1. **Phase diagram for the eigenenergy winding number ν.** The boundaries of the different phase are corresponding to the EPs.

We find $f(\tilde{k})$ enclose $E = 0$ one time and $v = 1$ when $\delta > 0$ and $v = -1$ when $\delta < 0$ based on the Residue theorem.

- When $\gamma > |\delta + \eta|$ and $\gamma < |\delta - \eta|$, $E^{\pm}(k)$ have one crossing point with imaginary axes at $k = 3\pi/2$ and one crossing point with real axes at $k = \pi/2$. Still, they together form one closed loop in the complex plane. We can define a 4π-periodic piecewise function $g(\tilde{k})$ describing the continuous loop:

$$g(\tilde{k}) = \begin{cases} \sqrt{(\delta\cos\tilde{k} - i\gamma)^2 + (\delta\sin\tilde{k} + \eta)^2} & \tilde{k} \in \left[0, \frac{\pi}{2}\right) \\ -\sqrt{(\delta\cos\tilde{k} - i\gamma)^2 + (\delta\sin\tilde{k} + \eta)^2} & \tilde{k} \in \left[\frac{\pi}{2}, \frac{5\pi}{2}\right) \\ \sqrt{(\delta\cos\tilde{k} - i\gamma)^2 + (\delta\sin\tilde{k} + \eta)^2} & \tilde{k} \in \left[\frac{5\pi}{2}, 4\pi\right) \end{cases} \quad \text{(S16)}$$

We find $f(\tilde{k})$ enclose $E = 0$ one time and $v = 1$ when $\delta < 0$ and $v = -1$ when $\delta > 0$.

The sign of the eigenenergy winding number determines the direction of complex band windings. The phase transition occurs at the EPs. The phase diagram is shown in Fig. S1.

B. Phase diagram of eigenstate winding numbers

We can define the topological phase of the non-Hermitian SSH mode. Under periodic boundary condition, the bulk spectrum in the momentum space takes the form of a 2×2 matrix

$$\hat{H}(k) = \begin{pmatrix} 0 & E_1(k) \\ E_2(k) & 0 \end{pmatrix}, \tag{S17}$$

where $E_1(k) = -\delta - i\,(\eta - \gamma)\, e^{-ik}$ and $E_2(k) = -\delta + i\,(\eta + \gamma)\, e^{ik}$. The Hamiltonian has the sublattice symmetry $\mathcal{S}^{-1} H(k) \mathcal{S} = -H(k)$, with $\mathcal{S} = \sigma_z$. As a result, the winding number of the system is given by[10]

$$\begin{aligned} w &= \int_0^{2\pi} \frac{dk}{4\pi i} Tr \left[\sigma_z \hat{H}^{-1}(k) \frac{\partial}{\partial_k} \hat{H}(k) \right] \\ &= \int_0^{2\pi} \frac{dk}{4\pi i} Tr \left[\sigma_z \frac{1}{E_1 E_2} \begin{pmatrix} 0 & E_1 \\ E_2 & 0 \end{pmatrix} \begin{pmatrix} 0 & \partial_k E_1 \\ \partial_k E_2 & 0 \end{pmatrix} \right] \\ &= \int_0^{2\pi} \frac{dk}{4\pi i} Tr [\begin{pmatrix} \frac{\partial_k E_2}{E_2} & 0 \\ 0 & -\frac{\partial_k E_1}{E_1} \end{pmatrix}] \\ &= \frac{1}{2} \left(\int_0^{2\pi} \frac{dk}{2\pi i} \frac{\partial_k E_2}{E_2} - \int_0^{2\pi} \frac{dk}{2\pi i} \frac{\partial_k E_1}{E_1} \right) \\ &\equiv \frac{1}{2} \left(\gamma_2 - \gamma_1 \right) \end{aligned} \tag{S18}$$

where

$$\gamma_1 = \frac{1}{2\pi i} \oint_{BZ} \frac{dE_1}{E_1}, \quad \gamma_2 = \frac{1}{2\pi i} \oint_{BZ} \frac{dE_2}{E_2} \tag{S19}$$

Geometrically, γ_1 (or γ_2) describes the winding number of a circle centering $E = \delta$ with radius $\eta - \gamma$ ($\eta + \gamma$). As a result, it's easy to see

$$\begin{cases} |\delta| < |\eta - \gamma| & \gamma_1 = -1 \\ |\delta| > |\eta - \gamma| & \gamma_1 = 0, \end{cases} \tag{S20}$$

and

$$\begin{cases} |\delta| < |\eta + \gamma| & \gamma_2 = 1 \\ |\delta| > |\eta + \gamma| & \gamma_2 = 0. \end{cases} \tag{S21}$$

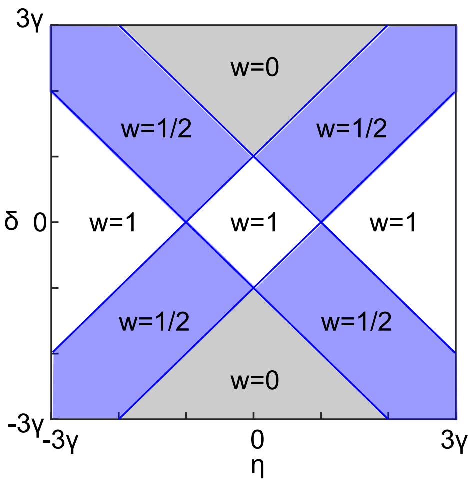

Figure S2. **Phase diagram for the eigenstate winding numbers w.** The boundaries of the different phase are corresponding to the EPs.

Then the winding number for the model is given by

$$\begin{cases} |\delta| < |\eta+\gamma|, |\eta-\gamma| & w=1 \\ |\delta| > |\eta+\gamma|, |\eta-\gamma| & w=0 \\ |\delta| < |\eta+\gamma|, \delta > |\eta-\gamma| & w=\frac{1}{2} \\ |\delta| > |\eta+\gamma|, \delta < |\eta-\gamma| & w=\frac{1}{2}. \end{cases} \tag{S22}$$

C. Phase diagram of the tight binding semi-infinity lattice

Starting from Eq. (S8), we review some bulk characters of the 1D tight binding non-Hermitian lattice. As we destroy the polarization couplings between the modes of $m=0$. The OAM lattice will split into two chains with sharp boundary condition. Here we focus on the edge state with zero eigenergies. For the edge state localized at the left edge on the right chain, the wave function takes the form of

$$|\psi\rangle = (\psi_{\circlearrowleft,0}, \psi_{\circlearrowright,0}, \psi_{\circlearrowleft,1}, \psi_{\circlearrowright,1} \cdots, \psi_{n,\circlearrowleft}, \psi_{n,\circlearrowright}, \cdots). \tag{S23}$$

Then we use the ansatz for the above wave function:

$$\psi_{\circlearrowleft,n} = \psi_{\circlearrowleft,0} z^n \quad \psi_{\circlearrowright,n} = \psi_{\circlearrowright,0} z^n. \tag{S24}$$

Apply the ansatz to the eigenstate equation $H|\psi\rangle = 0$, we find

$$\begin{aligned} &[\delta - i(\eta+\gamma)z]\psi_{\circlearrowleft,0} = 0 \\ &[i(\eta-\gamma) + \delta z]\psi_{\circlearrowright,0} = 0. \end{aligned} \tag{S25}$$

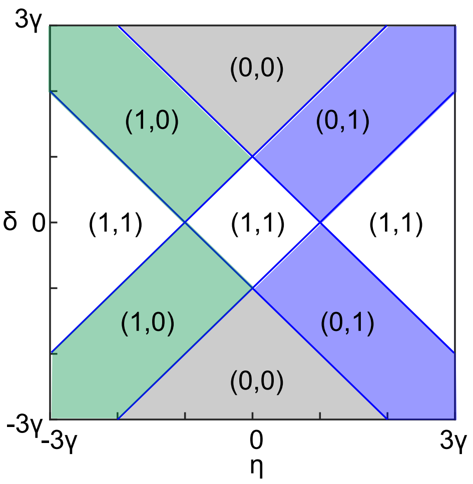

Figure S3. **Phase diagram for the boundary states.** $(1,0)$ represents the boundary states locate on the left edge of the right chain; $(0,1)$ represents the boundary states locate on the right edge of the left chain; $(1,1)$ represents both boundary states locates both on the left and right edges of the two chains; $(0,0)$ represents none boundary state;

As a result, the solution is given by:

$$z = \frac{\delta}{i(\eta+\gamma)} \quad \psi_{\circlearrowright,0} = 0, \tag{S26}$$

which exists when

$$|\frac{\delta}{i(\eta+\gamma)}| < 1 \Rightarrow |\delta| < |\eta+\gamma|. \tag{S27}$$

Similarly, for the eigenstate at the wight edge of the right chain, the wave function takes the form of

$$|\psi\rangle = (\psi_{\circlearrowright,-1}, \psi_{\circlearrowleft,-1}, \psi_{\circlearrowright,-2}, \psi_{\circlearrowleft,-2}, \cdots, \psi_{\circlearrowright,-n}, \psi_{\circlearrowleft,-n}, \cdots). \tag{S28}$$

Then we use the ansatz for the above wave function:

$$\psi_{\circlearrowright,-n} = \psi_{\circlearrowright,-1} z^{n-1} \quad \psi_{\circlearrowleft,-n} = \psi_{\circlearrowleft,-1} z^{n-1}. \tag{S29}$$

Apply the ansatz to the eigenstate equation $H|\psi\rangle = 0$, we find

$$\begin{aligned} &[\delta - i(\eta-\gamma)z]\psi_{\circlearrowright,-1} = 0 \\ &[i(\eta+\gamma) + \delta z]\psi_{\circlearrowleft,-1} = 0. \end{aligned} \tag{S30}$$

As a result, the solution is given by:

$$z = \frac{\delta}{i(\eta-\gamma)} \quad \psi_{\circlearrowright,-1} = 0, \tag{S31}$$

which exists when

$$|\frac{\delta}{i(\eta-\gamma)}| < 1 \Rightarrow |\delta| < |\eta - \gamma|. \tag{S32}$$

The phase diagram of the boundary states is shown in Fig. S3. Both of the eigenstate winding numbers w and eigenenergy winding number v determines the boundary states.

III. EXPERIMENTAL DETAILS

A. Input and output relations of the cavity

Using a specific OAM mode to drive the degenerate cavity with a specific cavity length, the corresponding lattice site can be excited. In order to obtain the interaction information of modes in the degenerate cavity, it is necessary to detect the output signals of the cavity.

Here we consider the input light field as $|in\rangle = |s_{in}\rangle \otimes |\mathrm{O}_{in}\rangle$, where $|s_{in}\rangle$ and $|\mathrm{O}_{in}\rangle$ represent the initial polarization and OAM states, respectively. As passes through the first cavity mirror, part of the photons are coupled into the cavity, and the state is expressed as $\kappa|in\rangle$, where κ is the coupling coefficient of the first cavity mirror. The output state $|out\rangle$ is contributed by the coherent transmitted light field for each round trip (labeled by l). Assuming the second cavity mirror has a coupling coefficient, the output state satisfies

$$|out\rangle = -|\kappa|^2 \sum_l t^l e^{il\beta\Delta L} U^l |in\rangle, \tag{S33}$$

where $\Delta L = mod(L, \lambda)$. L is the length of the cavity and λ is the wavelength of the photons. t represents the reflectivity of the cavity mirror, satisfying $|\kappa|^2 + |t|^2 = 1$. $\beta = 2\pi/\lambda$ is the wave number. $U = e^{-iH}$ is the unitary operator and H is the Hamiltonian, which is dependent on the optical elements inside the cavity. For brevity, we set

$$H = \begin{pmatrix} 0 & E_1 \\ E_2 & 0 \end{pmatrix}, \tag{S34}$$

and the unitary operator is

$$U = \begin{bmatrix} \cos(\sqrt{E_1E_2}) & -i\sqrt{\frac{E_1}{E_2}}\sin(\sqrt{E_1E_2}) \\ -i\sqrt{\frac{E_2}{E_1}}\sin(\sqrt{E_1E_2}) & \cos(\sqrt{E_1E_2}) \end{bmatrix} \tag{S35}$$

The eigenenergies are $E_\pm = \pm\sqrt{E_1E_2}$, and right and left eigenstates are $|R_\pm\rangle$ and $|L_\pm\rangle$, which satisfy

$$H\,|R_\pm\rangle = E_\pm\,|R_\pm\rangle \text{ and } \langle L_\pm|\,H = \langle L_\pm|\,E_\pm, \tag{S36}$$

where the eigenstates satisfy $\langle L_s \mid R_{s'}\rangle = \delta_{ss'}$ and $\sum_s |R_s\rangle\langle L_s| = I$ ($s = \pm$ and $s' = \mp$). So the eigenstates are

$$|R_\pm\rangle = \alpha \begin{bmatrix} \pm\sqrt{\frac{E_1}{E_2}} \\ 1 \end{bmatrix}, \qquad \langle L_\pm| = \frac{1}{2\alpha}\begin{bmatrix} \pm\sqrt{\frac{E_2}{E_1}} & 1 \end{bmatrix}, \tag{S37}$$

where α is an arbitrary factor. As we project the output photons on different OAM modes $|m\rangle\langle m|$, we can obtain the total evolution results of l-step as excited the site, where the intensity distribution denotes as

$$\begin{aligned} I(m, \beta\Delta L) &= \langle out|m\rangle\langle m|out\rangle \\ &= |\kappa|^4(|\sum_l t^l e^{il\beta\Delta L}\langle m|U^l|in\rangle|^2. \end{aligned} \tag{S38}$$

This intensity (probability) distribution can directly reveal the evolutionary direction influenced by the skin effect.

On the other hand, the output state can be expressed as

$$\begin{aligned} |out\rangle &= -|\kappa|^2 \sum_l t^l e^{il\beta\Delta L} U^l |s_{in}\rangle |\mathrm{O}_{in}\rangle \\ &= -|\kappa|^2 \sum_l t^l e^{il\beta\Delta L} U^l \sum_s |R_s\rangle\langle L_s| \sum_k |k\rangle\langle k|s_{in}\rangle|\mathrm{O}_{in}\rangle \\ &= -|\kappa|^2 \sum_{k,s}\sum_l t^l e^{il\beta\Delta L} e^{-ilE_s(k)}\langle L_s, k \mid s_{in}, \mathrm{O}_{in}\rangle |R_s, k\rangle \\ &= -|\kappa|^2 \sum_{k,s} \frac{1}{1 - te^{i[\beta\Delta L - E_s(k)]}}\langle L_s, k \mid s_{in}, \mathrm{O}_{in}\rangle |R_s, k\rangle \\ &\approx -\frac{|\kappa|^2}{t}\sum_k \frac{1}{\Gamma - \mathrm{Im}E(k) + i[\beta\Delta L - \mathrm{Re}E(k)]}\langle L_s, k \mid s_{in}, \mathrm{O}_{in}\rangle |R_s, k\rangle \end{aligned} \tag{S39}$$

where $\Gamma = 1/t - 1 + \gamma$ represents the loss of the cavity. By setting the input state to be $|s_{in}\rangle = |\circlearrowleft\rangle$ (or $|\circlearrowright\rangle$) and $|\mathrm{O}_{in}\rangle = |0\rangle$, the output intensity $I_{out} = \langle out|\circlearrowleft\rangle\langle\circlearrowleft|out\rangle$ (or $\langle out|\circlearrowright\rangle\langle\circlearrowright|out\rangle$) can be denoted as

$$I_{all}(\beta\Delta L) \propto \sum_k |G|^2 = \sum_k \frac{1}{[\Gamma - \mathrm{Im}E(k)]^2 + [\beta\Delta L - \mathrm{Re}E(k)]^2}, \tag{S40}$$

where $G = \{\Gamma - \mathrm{Im}E(k) + i[\beta\Delta L - \mathrm{Re}E(k)]\}^{-1}$ represents the Green function of the system. The output light field contains all of the k components, which reveals the density of the states (DOS). Furthermore, the k-resolved output light field intensity $I_k = \langle out|k\rangle\langle k|out\rangle$ can be written as

$$I(k, \beta\Delta L) \propto |G|^2 = \frac{1}{[\Gamma - \mathrm{Im}E(k)]^2 + [\beta\Delta L - \mathrm{Re}E(k)]^2}. \tag{S41}$$

According to Eq. S41, we can extract the imaginary part and the real part of the energy. As we scan the cavity length ΔL, we can get a transmitted intensity distribution spectrum $I(k, \beta\Delta L)$. Only when $\Delta L =$

$\mathrm{Re}E(k)/\beta$ the transmission intensity reaches the local maximum (peak, $I_{\max}(k)$), and we can determine the real part of the energy as $\mathrm{Re}E(k) = \Delta L\beta$. The local maximum satisfies $I_{\max}(k) \approx \Gamma_0^2[\Gamma - \mathrm{Im}E(k)]^{-2}$, where $\Gamma_0 = 1/t - 1$, and we can determine the imaginary part of the energy as $\mathrm{Im}E(k) \approx \Gamma - \Gamma_0/\sqrt{I_{\max}(k)}$. Worthy to note the imaginary part of the energy is more accurate as the imaginary energy is farther away from EPs.

To detect the eigenstate winding number, we need to further apply polarisation projective measurement on the output photons. The input polarization state is prepared as $|s_{in}\rangle = |\circlearrowright\rangle$ and project the output states on the basis of $\sigma_x = |H\rangle\langle H| - |V\rangle\langle V|$, where $|H\rangle = \frac{|\circlearrowright\rangle + |\circlearrowleft\rangle}{\sqrt{2}}$ and $|V\rangle = \frac{|\circlearrowright\rangle - |\circlearrowleft\rangle}{\sqrt{2}}$. The projective intensity of the corresponding band (here we chose the band index $s = +$ without loss of generality) can be written as

$$
\begin{aligned}
I_x(k) &= I_{x_+}(k) - I_{x_-}(k) \\
&= I_{\max}(k)\frac{1}{4\alpha^2}\left(|\langle H|R_+\rangle|^2 - |\langle V|R_+\rangle|^2\right) \\
&= I_{\max}(k)\left(|(1+\sqrt{E_1/E_2})|^2 - |(1-\sqrt{E_1/E_2})|^2\right) \\
&= I_{\max}(k)\,\mathrm{Re}[\sqrt{E_1/E_2}],
\end{aligned} \tag{S42}
$$

here we can define the $n_x(k) = I_x(k)/I_{\max}(k) = \mathrm{Re}[\sqrt{E_1/E_2}]$. Similarly, we can project the output state on σ_y and σ_z to get $n_y(k) = \mathrm{Im}[\sqrt{E_1/E_2}]$ and $n_z(k) = 0$, where $\sigma_y = |A\rangle\langle A| - |D\rangle\langle D|$ and $\sigma_z = |\circlearrowright\rangle\langle\circlearrowright| - |\circlearrowleft\rangle\langle\circlearrowleft|$. The projective basis are $|A\rangle = \frac{|\circlearrowright\rangle - i|\circlearrowleft\rangle}{\sqrt{2}}$ and $|D\rangle = \frac{|\circlearrowright\rangle + i|\circlearrowleft\rangle}{\sqrt{2}}$. The eigenstate winding number can be represented by the vector $[n_x(k), n_y(k)]$, denoted as

$$
\begin{aligned}
w &= \int_0^{2\pi}\frac{dk}{4\pi i}\,\mathrm{Tr}\left[\sigma_z\hat{H}^{-1}(k)\frac{\partial}{\partial_k}\hat{H}(k)\right] \\
&= -\frac{1}{2\pi i}\oint_{BZ} d\left(\ln\sqrt{\frac{E_1}{E_2}}\right) \\
&= -\frac{1}{2\pi}\oint_{BZ} d\left(\arg\left[\sqrt{\frac{E_1}{E_2}}\right]\right) \\
&= -\frac{1}{2\pi}\oint_{BZ} d\left(\arg\left[n_x + i n_y\right]\right).
\end{aligned} \tag{S43}
$$

Thus, we can simply plot the $[n_x(k), n_y(k)]$ $k \in \{0, 2\pi\}$ in a plane, and the eigenstate winding number can be directly obtained.

B. Details of the experimental settings

The detailed experimental setup is shown in Fig. S4. A Gaussian infrared continuous wave (CW) laser with the wavelength at $\lambda = 880$ nm is used to pump the cavity to excite the lattice site $n = 0$. Though a

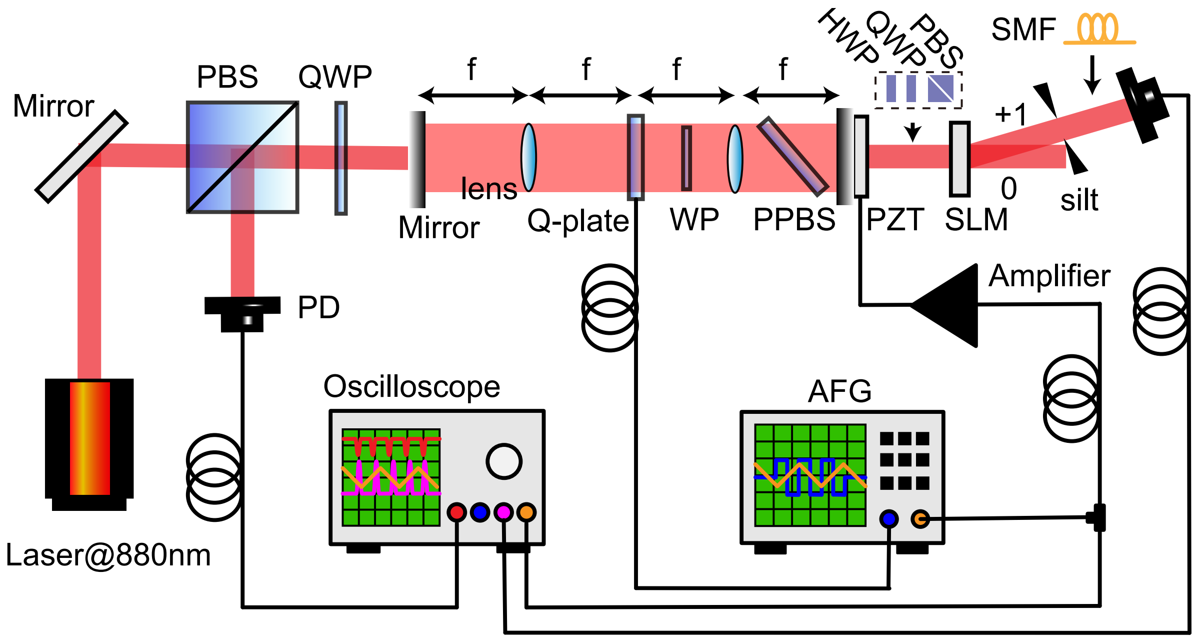

Figure S4. **Schematic diagram of the experimental setup.** PD: photodetector; AFG: arbitrary function generator; WP: wave plate; PPBS: partially polarized beam splitter; PBS: polarized beam splitter; PZT: piezoelectric transducer; SLM: liquid crystal spatial light modulator; SMF: single mode fiber; HWP: half wave plate; QWP: quarter-wave plate; PBS: polarized beam splitter.

polarization beam splitter (PBS) and a quarter-wave plate (QWP) with the optical-axis setting at $\pm 45°$, the polarisation can be prepared to left or right circular ($\circlearrowleft$ or $\circlearrowright$) to excite specific spin.

A Q-plate with $q = 1$ is placed in the confocal plane of two lenses in the cavity, of which the parameter δ can be adjusted by an additional electrostatic field supplied via an arbitrary function generator (AFG). A $3\lambda/2$-wave plate (WP) is set behind the Q-plate with the optical axis set to be $\pm 90°$ to control the skin effect directions. A partially polarized beam splitter (PPBS) is placed to introduce unbalance loss, where the transmittance is about 50% for the vertically polarized photons ($\frac{|\circlearrowleft\rangle - |\circlearrowright\rangle}{\sqrt{2}}$) while the transmittance is 99.9% for the horizontally polarized photons ($\frac{|\circlearrowleft\rangle + |\circlearrowright\rangle}{\sqrt{2}}$). A piezoelectric transducer (PZT) is pasted on the output mirror to scan the cavity length ΔL, which is driven by an amplified periodic triangular wave signal generated by the AFG. The AFG connects with an oscilloscope with a 1 GHz bandwidth to use the triangular wave signal as a trigger Level reference. The scanning frequency is about 40 Hz, which is much less than the FSR of the cavity. Thus, the cavity reaches steady at each length. The max scanning length is greater than 1 μm to ensure the scan cavity detuning covers the FSR of the cavity and obtains the full energy spectrum.

The uncoupled photons are reflected by the PBS and detected by a photodetector (PD). The PD connects the oscilloscope to display the reflected signal as a reference to sort the transmitted spectra. The transmitted

light is modulated by a liquid crystal spatial light modulator (SLM) loaded with different phase holograms followed by a silt or an SMF for OAM or k projective measurements. A half-wave plate (HWP), a quarter-wave plate (QWP), and a polarized beam splitter (PBS) are set for polarisation projective measurements to get eigenstate winding numbers. All the signals detected by PDs are displayed on the oscilloscope to read the energy spectrum.

C. Modification of the experimental eigenstate winding number

When the parameters δ and η are not small enough, the system does not satisfy the tight-binding approximation. For the periodic-driven nature of our cavity, we can describe the system using the Floquet theory. Like the processing method in Ref.[31], we define two time frames of the Floquet system to investigate the symmetry and topology properties, where the evolutions satisfy

$$\begin{aligned} U_1 &= J_Q J_W J_M J_M J_W J_Q, \\ U_2 &= J_M J_W J_Q J_Q J_W J_M. \end{aligned} \tag{S44}$$

Both of the two time frames have the sublattice symmetry as

$$\mathcal{S} U_\alpha(k) \mathcal{S} = U_\alpha^{-1}(k), \quad \alpha = 1, 2. \tag{S45}$$

Where $\mathcal{S} = \sigma_z$. According to $U_\alpha = e^{-iH_\alpha}$ and the definition of the eigenstate winding number is

$$\begin{aligned} W_\alpha &= \int_0^{2\pi} \frac{dk}{4\pi i} Tr\left[\sigma_z \hat{H}_\alpha^{-1}(k) \frac{\partial}{\partial_k} \hat{H}_\alpha(k)\right] \\ &= \frac{1}{2}\left(\frac{1}{2\pi i}\oint_{BZ} d\ln\frac{U_\alpha^{2,1}}{U_\alpha^{1,2}}\right), \end{aligned} \tag{S46}$$

where $U_\alpha^{1,2}$ and $U_\alpha^{2,1}$ represent the antiangular matrix elements of U_α. The pair of the topological invariants for the gap at 0 and π are given by the average value of the winding number in the two frameworks

$$w_0 = \frac{W_1 + W_2}{2}, \quad w_\pi = \frac{W_1 - W_2}{2}. \tag{S47}$$

In our settings of $\eta = -0.5\pi$, the winding number is always $W_2 = 0$ for the time frame 2. So we only detect the winding number W_1 in the time frame 1 and get the invariants $w_0 = W_1/2$ for the zero-energy edge states.

On the other hand, the choice of the unit cells will also cause the different topological winding numbers. In experiments, we detect the winding number w_{exp} based on the unit cells in Fig. S5a. The ping hole cut off the lattice and the breakpoint living within the unit cell. In order to make the winding number correspond to

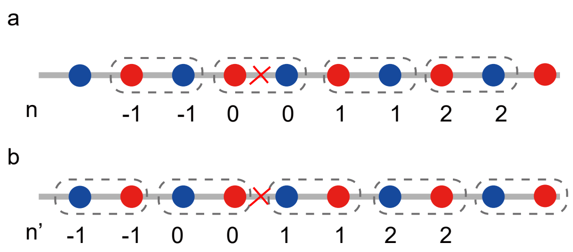

Figure S5. Shift of the unit cells. a. The experimental unit cells; b. The theoretical unit cells.

the boundary state, we need to shift the unit cell as shown in Fig. S5b. This shift will introduce two phases in the nondiagonal term of a Hamiltonian as

$$H = \begin{pmatrix} 0 & E_1 e^{-ik} \\ E_2 e^{ik} & 0 \end{pmatrix}. \tag{S48}$$

So the eigenstate winding number should be changed to

$$\begin{aligned} w &= -\frac{1}{2\pi i}\oint_{BZ} d\left(\arg\left[\sqrt{\frac{E_1 e^{-ik}}{E_2 e^{ik}}}\right]\right) \\ &= -\frac{1}{2\pi i}\oint_{BZ} d\left(\arg\left[\sqrt{\frac{E_1}{E_2}}\right]\right) + 1 \\ &= w_0 + 1. \end{aligned} \tag{S49}$$

Considering both Floquet modification and unit cell shift, the desired winding number should modified to

$$w = w_0 + 1 = \frac{W_1}{2} + 1. \tag{S50}$$

IV. SEMI-INFINITE CHAIN APPROXIMATION

In principle, the number of OAM modes is unbounded. The transverse profiles of these modes expand with increasing OAM number, at a diminishing rate for large OAM values, with the radius scaling as $\sqrt{n}$. If the aperture of the optical elements in our experimental setup were infinitely large, an ideal semi-infinite lattice could be realized. However, in practice, optical components have finite dimensions. First, we examine the impact of finite-size mirrors. As photons propagate toward high-OAM sites, they increasingly escape the cavity without being reflected back, leading to a lack of interference with photons near $n = 0$. Consequently, the edge states exhibit behavior analogous to that observed in a semi-infinite chain. This effect can be effectively modeled as on-site losses, which eventually saturate at a value approximately equal to the free spectral range (FSR), beyond which photons escape the cavity within a single round trip. Next,

we consider the influence of the Q-plate, WP, and PPBS. If these elements were infinitely large, tunneling amplitudes between OAM modes would remain constant across the lattice. However, in a realistic scenario with finite-sized elements, tunneling rates decrease as the OAM number increases. Without loss of generality, we analyze the right-partitioned OAM lattice (Fig. S6a). The system Hamiltonian can be expressed as

$$
\begin{aligned}
H = &-\sum_{n=0}^{\infty} J_n \left\{ \delta(a^{\dagger}_{\circlearrowright,n} a_{\circlearrowleft,n} + \text{h.c.}) + i \left[(\eta+\gamma) a^{\dagger}_{\circlearrowright,n} a_{\circlearrowleft,n+1} - (\eta-\gamma) a^{\dagger}_{\circlearrowleft,n+1} a_{\circlearrowright,n} \right] \right\} \\
&-\sum_{n=0}^{\infty} i\Gamma_n (a^{\dagger}_{\circlearrowleft,n} a_{\circlearrowleft,n} + a^{\dagger}_{\circlearrowright,n} a_{\circlearrowright,n}),
\end{aligned}
\tag{S51}
$$

The coefficients J_n (tunneling rates) begin to decrease, while Γ_n (on-site losses) increase as the OAM number becomes sufficiently large. As $n \to \infty$, J_n asymptotically approaches zero, whereas Γ_n saturates at a finite value. Notably, the system lacks a well-defined sharp boundary at the high-OAM end. Consequently, the non-Bloch theory, which is formulated under the assumption of sharp boundaries, is not applicable in this scenario.

We can demonstrate that the zero-energy edge mode at $n = 0$ in our system is equivalent to that of a semi-infinite lattice. To understand this, let's first consider a finite (N unit cells) open SSH lattice with sharp boundaries. We can solve for the two edge modes approximately by assuming semi-infinite boundary conditions, and investigate the exact solutions of a finite lattice perturbatively. The two edge-mode solutions read $\psi_{\text{left}} = \chi_{\circlearrowleft} e^{-n/\xi_t}$ and $\psi_{\text{right}} = \chi_{\circlearrowright} e^{(n-N)/\xi_t}$ with $\chi_{\circlearrowleft}$ and $\chi_{\circlearrowright}$ the polarization wave functions, ξ_t the topological localization length. For a finite lattice, the two degenerate modes couple with each other, with coupling rate exponentially small. The two solutions are hardly affected by the far-detuned bulk modes. Within the edge-mode subspace, the effective Hamiltonian reads

$$
H_{\text{eff}} \sim \begin{bmatrix} 0 & e^{-N/\xi_t} \\ e^{-N/\xi_t} & 0 \end{bmatrix}
\tag{S52}
$$

Therefore, the eigen energy and state are $E_\pm \sim \pm e^{-N/\xi_t}$ and $\psi_\pm \sim \psi_{\text{left}} \pm \psi_{\text{right}}$. In the presence of skin effect (assuming towards the right edge), the two eigen solutions become $\psi_\pm \sim e^{-N/\xi_s} \psi_{\text{left}} \pm \psi_{\text{right}}$ with ξ_s the localization length of the skin effect. We see that both the two eigen solutions located at the right edge with negligible distributions on the left edge.

Then we turn to our case and consider a large cutoff N for the lattice sites. First we ignore the skin effects (e.g., by the similarity transformation). The approximate edge-mode solution at the left edge remains the same. However, the approximate edge-mode solution around the right edge no longer have zero energy due

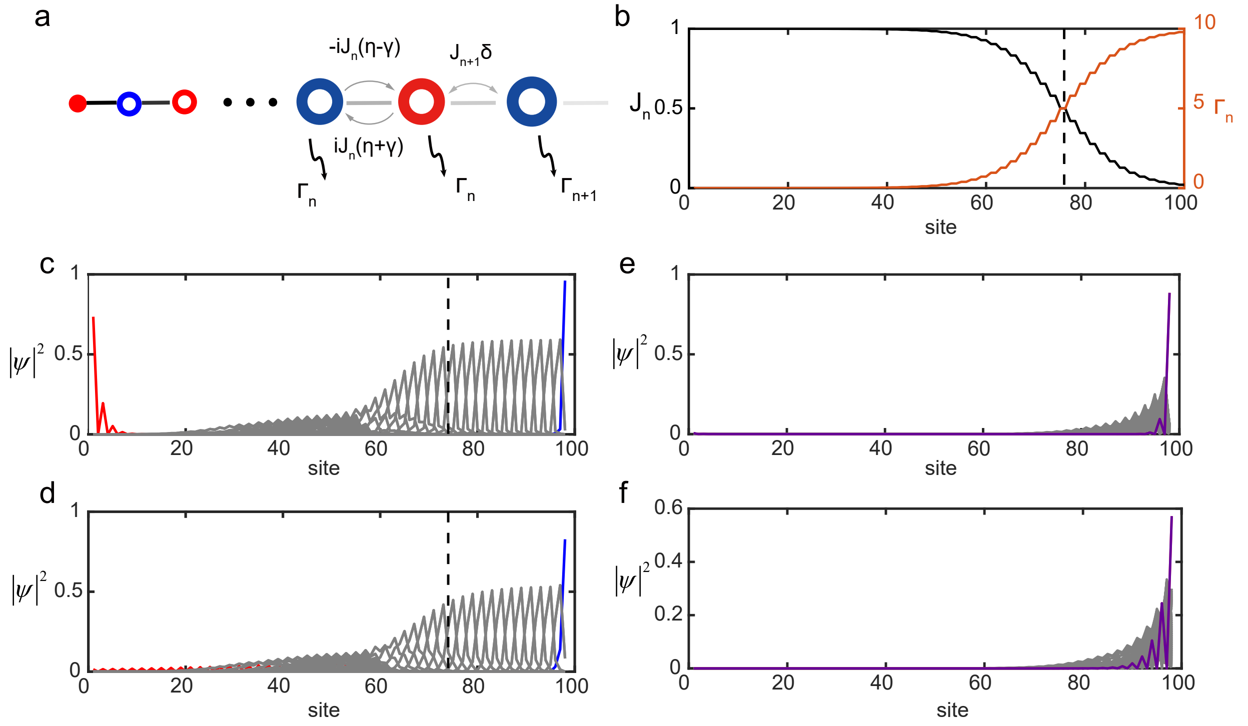

Figure S6. **a** Schematic representation of the lattice under soft-edge conditions. **b** The distribution of the tunneling coefficient J_n and the on-site loss Γ_n. The dotted line indicates the center of the soft edge, determined by the aperture of the cavity system. **c-d** Eigenstate distributions under soft-edge conditions for $\delta = 0.2\pi$ (**c**) and $\delta = 0.4\pi$ (**d**). **e-f** Eigenstate distributions under sharp open boundary conditions for $\delta = 0.2\pi$ (**e**) and $\delta = 0.4\pi$ (**f**). Edge states are highlighted in red and blue, while bulk states are shown in gray. The other parameters are set as $\eta = -0.5\pi$ and $\gamma = 0.35$.

to the large on-site loss, the effective Hamiltonian becomes

$$H_{\text{eff}} \sim \begin{bmatrix} 0 & e^{-N/\xi_t} \\ e^{-N/\xi_t} & -i\Gamma_N \end{bmatrix} \tag{S53}$$

The eigen energies become $E_\pm = -i\Gamma_N/2 \pm i\sqrt{\Gamma_N^2/4 - e^{-2N/\xi_t}} \simeq -i\Gamma_N/2 \pm (i\Gamma_N/2 - ie^{-2N/\xi_t}/\Gamma_N)$, that is $E_+ = -ie^{-2N/\xi_t}/\Gamma_N \sim 0$ and $E_- = -i\Gamma_N - ie^{-2N/\xi_t}/\Gamma_N \sim -i\Gamma_N$. The eigen states (to the leading order) become $\psi_\pm \sim [e^{-N/\xi_t}, \pm i\sqrt{\Gamma_N^2/4 - e^{-2N/\xi_t}} - i\Gamma_N/2]$, that is $\psi_+ \sim \Gamma_N\psi_{\text{left}} - ie^{-N/\xi_t}\psi_{\text{right}}$ and $\psi_- \sim e^{-N/\xi_t}\psi_{\text{left}} - i\Gamma_N\psi_{\text{right}}$. Now taking into account of the skin effects, the solutions become

$$\psi_+ \sim \Gamma_N\psi_{\text{left}} - ie^{N/\xi_s}e^{-N/\xi_t}\psi_{\text{right}} \tag{S54}$$

$$\psi_- \sim e^{-N/\xi_s}e^{-N/\xi_t}\psi_{\text{left}} - i\Gamma_N\psi_{\text{right}} \tag{S55}$$

Now it is clear that we have a zero mode solution $\psi_+ \sim \psi_{\text{left}}$ located at the left edge as long as $\xi_t < \xi_s$. This edge mode disappears from the left edge at $\xi_t > \xi_s$, the transition point $\xi_t = \xi_s$ is just the EP point.

Notice that $1/\xi_t = \ln(\frac{\sqrt{\eta^2-\gamma^2}}{\delta})$ and $1/\xi_s = \ln(\sqrt{|\frac{\eta-\gamma}{\eta+\gamma}|})$, so $\frac{\sqrt{\eta^2-\gamma^2}}{\delta} = \sqrt{|\frac{\eta-\gamma}{\eta+\gamma}|}$ leads to $\delta = |\eta+\gamma|$. Here we assumed $\eta < 0$ such that $|\eta+\gamma| < |\eta-\gamma|$.

We have also numerically verified the aforementioned properties. Specifically, we simulate a 100-site (50 unit-cell) lattice with a soft boundary around the 75th site. The distributions of the tunneling coefficient J_n and the on-site loss Γ_n are depicted in Fig. S6b, exhibiting significant variations near the 75th site (indicated by the dotted line). The parameters are set to $\eta = -0.5\pi$ and $\gamma = 0.35$, consistent with the experimental configuration in Fig. 4. For $\delta = 0.2\pi$ ($\delta < |\eta+\gamma|$), the eigenstate distributions are presented in Fig. S6c. The bulk modes (gray) are spread across the entire soft boundary region, while two edge states are localized at the left (red) and right (blue) edges, respectively. Notably, the right edge state exhibits negligible resonance, as it is predominantly distributed outside the cavity. For $\delta = 0.4\pi$ ($\delta > |\eta+\gamma|$), the left edge state merges into the bulk, consistent with our experimental observations. These characteristics of the edge mode solutions are in agreement with those of a semi-infinite lattice.

For comparison, we compute the eigenstate distributions under sharp open boundaries using the same parameters, as illustrated in Fig. S6e ($\delta = 0.2\pi$) and Fig. S6f ($\delta = 0.4\pi$). In both cases, the two edge modes are confined to the right edge, consistent with the predictions of non-Bloch theory.

V. EXTENDED EXPERIMENTAL RESULTS

In this section, we conduct a examination of the eigenenergy windings with $v = 1$ and concurrently detect the corresponding edge states. The chosen parameters (γ, η, δ) are set at $(0.35, 0.5\pi, 0.4\pi)$. The transmitted intensity spectrum along k is presented in Fig. S7a, and the extracted complex bands are depicted in Fig. S7b. The two bands form a loop structure; however, the rotational direction of the loop changes from clockwise to anticlockwise as k spans from 0 to 2π compared to Fig. 2i, determining the eigenenergy winding number of $v = 1$.

The eigenenergy winding number indicates that the system is situated in the line-gap (gapless) phase. In this phase, the intensified left-toward skin effect eliminates the edge state in the left semi-infinite chain, leaving only one edge state positioned at the boundary of the tight semi-infinite chain (Fig. S7c). Subsequently, we detect the corresponding edge states on the two semi-infinite chains. The total transmission intensity spectrum when exciting modes on the left chain (via right-circular polarized laser pumping) is illustrated in Fig. S7d. Notably, there is no central peak in the gap, as it is ”dragged” away by the right-toward skin effect. Conversely, when exciting modes on the right chain (via left-circular polarized laser pumping), a strong peak emerges (Fig. S7e). This outcome emphasizes that the eigenenergy winding number v determines the side on which the edge states are located.

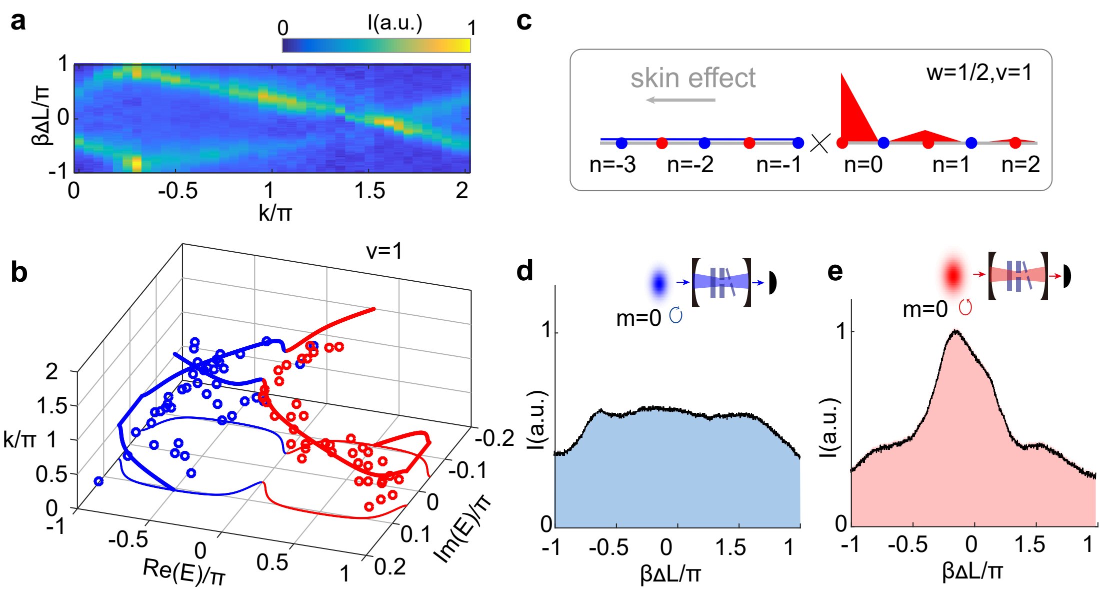

Figure S7. **Eigenenergy windings with $v = 1$ and it's edge state distribution.** **a**. The transmitted intensity spectra along momentum k when parameters (γ, η, δ) are $(0.35, 0.5\pi, 0.4\pi)$. **b**. The complex energies in $E-k$ space extracted from **a**. The circles represent the experimental data. The thick curves are the theoretical predictions, and the thin curves are the projections of the energies in the plane. **c**. Theoretical edge state distributions. The parameters (γ, η, δ) correspond to $(0.35, 0.5\pi, 0.4\pi)$. The eigenstate (w) and eigenenergy (v) winding numbers are labeled in the upper right corner. **d and e**. Total transmitted intensity spectra while pumping the cavity with the right (**b**) and left (**c**) -circular polarized Gaussian modes ($m = 0$), respectively.

---

* These authors contribute equally to this work

† luoxw@ustc.edu.cn

‡ jsxu@ustc.edu.cn

§ cfli@ustc.edu.cn
